# Poor Mental Health Status as a Risk Factor and Prognosticator in SMuRF-Less Acute Myocardial Infarction

**DOI:** 10.3390/jcm14082645

**Published:** 2025-04-11

**Authors:** Dimitrios V. Moysidis, Georgios Giannopoulos, Vasileios Anastasiou, Stylianos Daios, Andreas S. Papazoglou, Alexandros C. Liatsos, Efstathios Spyridonidis, Vasileios Kamperidis, Matthaios Didagelos, Georgios Tagarakis, Christos Savopoulos, Panagiotis Kyriakidis, Sonia Konstantinidou, George Giannakoulas, Vassilios Vassilikos, Antonios Ziakas

**Affiliations:** 1 Third Department of Cardiology, Hippokration General Hospital, Aristotle University of Thessaloniki, Konstantinoupoleos 49, 54642 Thessaloniki, Greece; 2 First Department of Cardiology, AHEPA University Hospital, Aristotle University of Thessaloniki, St. Kyriakidi 1, 54636 Thessaloniki, Greece; 3 424 General Military Hospital of Thessaloniki, 54621 Thessaloniki, Greece; 4 Cardiothoracic Surgery Department, AHEPA University Hospital, Aristotle University of Thessaloniki, St. Kyriakidi 1, 54636 Thessaloniki, Greece; 5 First Propedeutic Department of Internal Medicine, Aristotle University of Thessaloniki, St. Kyriakidi 1, 54636 Thessaloniki, Greece

**Keywords:** mental health status, mental component summary, coronary artery disease, acute myocardial infarction, standard modifiable risk factors

## Abstract

**Background:** The etiology of acute myocardial infarction (AMI) in patients without history of standard modifiable risk factors (SMuRFs) remains unclear. Simultaneously, evidence suggests that mental health status (MHS) contributes to the pathogenesis of AMI and worsens its outcomes. **Methods:** This analysis of the prospective “Beyond-SMuRFs” (NCT05535582) study included 650 consecutive patients with AMI who had available data on self-reported MHS before AMI, calculated by the SF36-Questionnaire mental component summary (MCS). Poor MHS was defined as MCS ≤ 50. Multivariable logistic-regression and Cox-regression analyses were implemented to investigate poor MHS as a potential predictor of SMuRF-less AMIs and compare all-cause mortality based on SMuRF-less and MH status, respectively. **Results:** Of 650 patients with AMI (mean age 62.6 ± 12.1 years), 288 (44.3%) had MCS ≤ 50 and 128 (19.7%) were SMuRF-less patients. Three out of four SMuRF-less patients reported an MCS ≤ 50 (n = 96, 75%), a significantly higher percentage than the corresponding percentage in patients with SMuRFs (n = 192, 36.8%; *p* < 0.01). The multivariable logistic regression model showed that MCS ≤ 50 was an independent predictor of SMuRF-less AMI [aOR = 0.95; 95% CI (0.94–0.96)]. Time-to-event analysis for all-cause mortality showed that patients with MCS > 50 had lower mortality rates than those with poor MHS (aHR, 3.61 [95% CI, 2.02 to 6.43], *p* < 0.01). Higher risk for all-cause mortality was also observed in SMuRF-less patients with poor MHS compared to patients with at least one SMuRF and good MHS [aHR, 4.52 (95% CI, 0.94–21.73)]. **Conclusions:** Poor MHS was an independent predictor of the occurrence of SMuRF-less AMI and predictive of higher mortality in patients with and without SMuRFs.

## 1. Introduction

Coronary artery disease (CAD) continues to be the leading cause of death globally [1]. Its incidence has been proven to be positively associated to the prevalence of standard modifiable cardiovascular risk factors (SMuRFs), such as diabetes mellitus, smoking, hypertension and dyslipidemia [2]. These comorbidities have been well recognized as precursors of atherogenesis and are utilized to evaluate the risk of sustaining an acute myocardial infarction (AMI). However, recent registries indicate a growing population of AMIs whose pathogenesis remains unclear, namely, those in patients without any SMuRF (SMuRF-less patients) [3,4,5]. Mental health status (MHS) seems to have a direct impact on cardiovascular health [6]. Patients with mental illness, such as major depression and schizophrenia, have been reported to have a higher prevalence of modifiable cardiovascular risk factors [7]. However, the association between mental status and cardiovascular disease appears to extend beyond SMuRFs, potentially involving as yet unelucidated genetic and epigenetic mechanisms [8,9].

Emerging evidence suggests that psychological distress can independently contribute to AMI risk, even in the absence of traditional cardiovascular risk factors. A growing body of literature has introduced the concept of “mental-stress-induced MI” (MSIMI), which refers to AMIs triggered by psychological stress rather than physical exertion [10,11]. MSIMI has been associated with various pathophysiological mechanisms, including endothelial dysfunction, microvascular dysfunction, enhanced smooth muscle reactivity, and dysregulated hypothalamic–pituitary–adrenal axis responses [12]. Additionally, the increased activation of brain regions involved in stress and pain processing, such as the prefrontal cortex, insula, and amygdala, has been implicated in the heightened cardiovascular risk observed in these patients [12]. These findings suggest that mental distress may contribute to AMI through complex neuroendocrine and inflammatory pathways, underscoring the need for further research into this underrecognized mechanism of cardiovascular disease.

To date, although evidence suggests that known and even underlying MHS disorders influence CAD development, the significance of mental status as a non-traditional predictor of SMuRF-less AMI has not been adequately investigated [13]. Large studies have shown higher morbidity and mortality rates in AMI patients who reported severe psychological distress [14,15]. Additionally, a recent meta-analysis of large observational studies demonstrated that SMuRF-less patients had higher mortality compared with patients with at least one traditional atherosclerotic risk factor [16]. However, there is a paucity of real-world data on the prognostic impact of poor mental status in AMI patients.

This study aimed to examine associations between mental health and SMuRF-less status by investigating the potential association of poor MHS with the occurrence of SMuRF-less AMIs. Secondarily, we aimed to assess the impact of mental status on future clinical outcomes of patients with AMI.

## 2. Methods

### 2.1. Study Design and Population

This study constitutes an analysis of the “Beyond-SMuRFs Study” (ClinicalTrials.gov Identifier: NCT05535582), a prospective, non-interventional cohort study involving patients with AMI undergoing coronary angiography at two academic tertiary hospitals and a military tertiary hospital in Thessaloniki, Greece [17]. The study was conducted in compliance with the fundamental principles set forth in the Declaration of Helsinki [18] and the guidelines of good clinical practice (GCP). It received approval from the Ethics Committee of Aristotle University of Thessaloniki (reference number: 136945/2022 [18]. All participants provided written informed consent before enrollment and special care was taken to comply with European directives and national law regarding data protection.

The design of the study, as well as detailed eligibility and exclusion criteria, was previously described [17]. Briefly, the registry of “Beyond-SMuRFs Study” aimed to investigate clinical and/or laboratory characteristics potentially associated with SMuRF-less AMIs by comparing the prevalence of clinical, laboratory and imaging parameters among patients with and without SMuRFs. The study included adult patients hospitalized for AMI with or without ST elevation within the previous 4 weeks and at least one stenosis >50% in a major epicardial coronary artery or a branch thereof with a diameter of at least 2 mm in coronary angiography. Patients with a history of previous AMI or previous coronary intervention, either percutaneous or surgical, patients over 80 years, and patients unable to provide informed consent were excluded from the registry. Patients with STEMI or Non-STEMI (NSTEMI), for whom baseline health status metrics (SF-36 Health Survey) and follow-up data were accessible, were included. Patients in the registry were divided into two groups based on their medical history. (a) Patients with SMuRFs, defined as those who fulfilled at least one of the following criteria: (i) self-reported use of tobacco products on a systematic basis for up to 12 months before AMI, (ii) known history of hypertension and/or antihypertensive treatment prior to AMI, (iii) known hypercholesterolemia (total cholesterol > 200 mg/dL/LDLc > 150 mg/dL) or treatment with statins or PCSK9is, before AMI, (iv) history of diabetes mellitus type 1 or 2 and/or treatment with antidiabetic tablets or insulin before AMI or diagnosis of diabetes mellitus based on HbA1c during AMI hospitalization. (b) SMuRF-less patients, who suffered an AMI in the total absence of these comorbidities.

All demographic, clinical, laboratory, imaging and medication data were obtained from the “Beyond-SMuRFs” database. Τhe following clinical and demographic characteristics were recorded in the registry for each patient: demographical data, socioeconomic parameters, medication and complete medical history, as well as prior diagnostic and therapeutic interventions. In addition, the 36-item short form (SF-36) standardized questionnaire was conducted to obtain a self-reported measure of patients’ health-related perceptions of quality of life before the AMI. Moreover, laboratory biomarkers of patients were recorded on admission and during hospitalization. A comprehensive evaluation was conducted, including a full blood count, routine biochemical markers, coagulation profile, thyroid function tests (TSH and thyroid hormones), HbA1c, NT-proBNP, and high-sensitivity troponin T (HsTnT) at the time of admission. Additionally, peak levels of NT-proBNP and HsTnT were recorded. Further assessments included measurements of lipoprotein(a) [LP(a)], apolipoproteins A1 and B (ApoA1 and ApoB), interleukin-6 (IL-6), and soluble urokinase plasminogen activator receptor (suPAR) at presentation. Coronary angiographic images were independently reviewed by interventional cardiologists, and a thorough transthoracic echocardiographic examination was performed within the first 24 h of hospitalization.

### 2.2. Definition of Covariates, Data Collection and Follow-Up

AMI was defined according to the Fourth universal definition of myocardial infarction [19]. Self-reported health-related perceptions of MHS during the weeks before AMI were evaluated during hospitalization according to the SF-36 Health questionnaire [20]. This is a standardized self-report questionnaire designed to evaluate health-related quality of life (QoL) across physical, mental, and social dimensions. The scoring ranges from 0 (worst) to 100 (best) and is based on 36 items covering eight health domains: physical functioning, limitations due to physical health issues, bodily pain, energy/fatigue, social functioning, limitations due to emotional difficulties, psychological distress, and overall well-being. These domains can be grouped into two main summary components—the Physical Component Summary (PCS) and the Mental Component Summary (MCS)—using an oblique model that allows for correlation between physical and mental health aspects [21].

**Figure 1:**
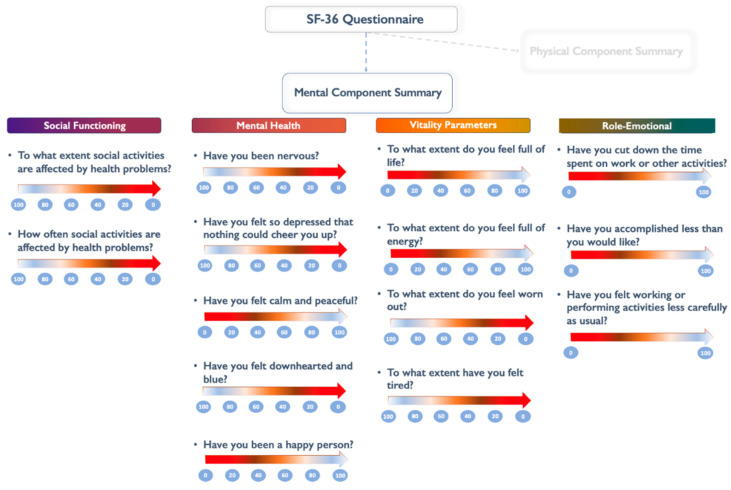
Graphical illustration of the SF-36 items included in MCS.

The MCS of the SF-36 questionnaire is derived from four subscales: Vitality (VT), Social Functioning (SF), Role–Emotional (RE), and Mental Health (MH). These subscales collectively assess different dimensions of mental well-being, including energy levels, limitations due to emotional problems, social interactions, and overall psychological distress or well-being. Each subscale is scored individually, and the MCS score is computed using a weighted algorithm that integrates these subscale scores into a composite measure of mental health (Figure 1). Patients were categorized into two groups based on the normalized population mean of 50 for the MCS in the SF-36 score—those with MCS ≤ 50 and those with MCS > 50. This approach has been utilized to streamline the interpretation of the SF-36 score in clinical settings. The questionnaire was performed the first day after coronary angiography. For the patients who were not able to provide reliable answers due to clinical status or death, a first-degree relative filled out the questionnaire.

Patients were also categorized based on their medical history into two groups. The first was (a) those with SMuRFs, identified as individuals meeting at least one of the following criteria: (i) a documented history of hypertension and/or prior use of antihypertensive medication before AMI, (ii) regular tobacco use within the 12 months preceding AMI, (iii) a history of type 1 or type 2 diabetes mellitus and/or prior treatment with antidiabetic medication or insulin, or a diabetes diagnosis based on HbA1c levels during AMI hospitalization, and (iv) known hypercholesterolemia (total cholesterol > 200 mg/dL or LDL-C > 150 mg/dL) or previous treatment with statins or PCSK9 inhibitors before AMI. The second was (b) SMuRF-less patients, defined as those who experienced an AMI without any of these underlying conditions.

All-cause mortality, defined as death from any cause, was the primary outcome of this study. Follow-up was performed by semi-annual clinic visits or phone communications with the patients themselves or their close relatives. Medical records were sought and reviewed if necessary. The first patient was enrolled in January 2022 and the follow-up process for this analysis was completed in September 2024.

### 2.3. Statistical Analysis

Baseline patient characteristics were examined by MHS (MCS ≤ 50 and MCS > 50). Patient characteristics were analyzed using the χ^2^ test for categorical variables and the two-sided Student’s *t*-test for continuous variables. When the assumption of normality was not met, the non-parametric Mann–Whitney U test was applied. Categorical variables are reported as frequencies and percentages (%), while continuous variables are presented as mean ± standard deviation (SD) or median (1st–3rd quartile).

Univariate logistic regression analysis was performed to identify significant predictors of SMuRF-less AMIs. A multivariable logistic regression model was then constructed by forcing univariably significant (*p* < 0.05) and clinically relevant variables into the multivariable model [age, sex, body mass index (BMI), glomerular filtration rate (GFR), history of psychiatric disorders, and history of diabetes mellitus]. Furthermore, a restricted cubic spline regression model was constructed to allow for plotting the adjusted odds ratios (ORs) for the prediction of SMuRF-less AMI along with the continuous range of MHS according to the MCS of the SF-36 score.

A time-to-event analysis was conducted to evaluate whether mental status was associated with better or worse clinical outcomes. Patients were censored at the time of the event or the last follow-up with the study investigator. Event rates were displayed using Kaplan–Meier (KM) curves, and comparisons were made using the Log-rank test. Multivariable Cox proportional hazards models were employed to adjust for baseline variables that were clinically relevant and univariately significant, including age, gender, BMI, GFR, acute heart failure at admission, and STEMI presentation. A two-tailed *p*-value of 0.05 was set as the threshold for statistical significance. All results were reported with 95% confidence intervals (CIs). Data management and statistical analyses were performed using SPSS software, version 26 (IBM SPSS Statistics) and R version 3.4.4 (R Foundation for Statistical Computing, Vienna, Austria).

## 3. Results

A total of 650 patients with AMI (STEMI or NSTEMI) were included in this study (mean age 62.6 ± 12.1 years). Of these, 288 (44.3%) patients had MCS ≤ 50, defined as poor mental status. Baseline patient characteristics according to SF-36 MCS are summarized in Table 1. In general, no significant differences were detected regarding demographics, past medical history or medication. Of 650 patients, 128 (19.7%) were SMuRF-less and 522 (80.3%) had at least one SMuRF.

**Figure 2:**
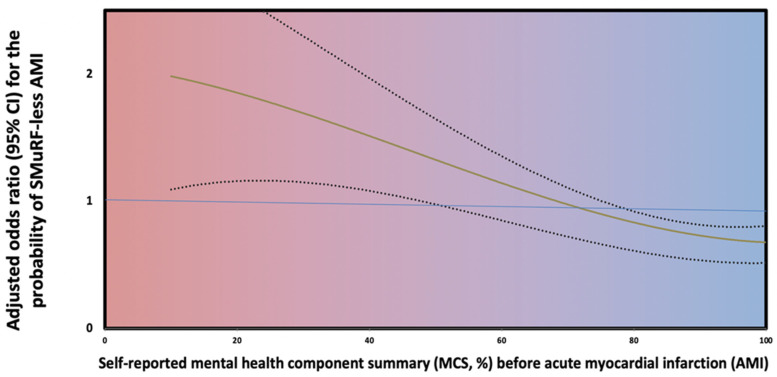
Correlation between the self-reported mental health status score (x axis) before AMI and the adjusted odds ratio (95% CI) for the probability of SMuRF-less AMI (y axis). The color scale corresponds to a gradual reduction in self-reported MCS scoring (from blue to red). These spline curves represent the adjusted odds ratios for the probability of a patient presenting with AMI being SMuRF-less depending on self-reported MCS. The edges of the darker area display 95% confidence intervals (CIs).

A multivariable logistic regression model was constructed to assess potential predictors of SMuRF-less AMI. After adjustments for univariately significant and clinically relevant parameters, it was found that self-reported MHS before AMI, as assessed by MCS, was an independent predictor of SMuRF-less AMI [aOR = 0.95; 95% CI (0.94–0.96)]. The spline curve graphically presenting the correlation between SF-36 MCS and aOR for the prediction of a SMuRF-less AMI is depicted in Figure 2. Three out of four (n = 96, 75%) SMuRF-less patients reported an SF-36 MCS of ≤50, which is significantly higher than the corresponding percentage in patients with SMuRFs (n = 192, 36.8%; *p* < 0.01).

**Figure 3:**
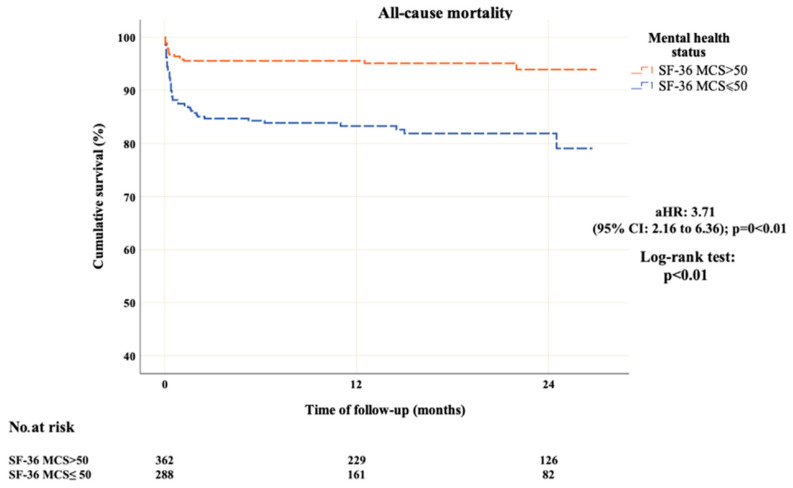
Time-to-event analysis for all-cause mortality in each SF-36 MCS subgroup.

Overall, 68 (10.5%) patients died over a median follow-up of 13 months [interquartile range (IQR): 5.6 to 20.4 months]. Of these, 54 (79.4%) died during the first 30-days of follow-up. Of the 68 patients, 50 (73.5%) had a below-average self-reported mental status before admission, while 18 patients (26.5%) had a self-reported SF-36 >50% [unadjusted hazard ratio (HR): 3.71] (95% CI: 2.16 to 6.36; *p* < 0.01 by the Log-rank test). Time-to-event analysis for all-cause mortality in each SF-36 MCS subgroup is exhibited in Figure 3. After adjustment, the hazards ratio (aHR) was 3.61 (95% CI: 2.02 to 6.43; *p* < 0.01).

**Figure 4:**
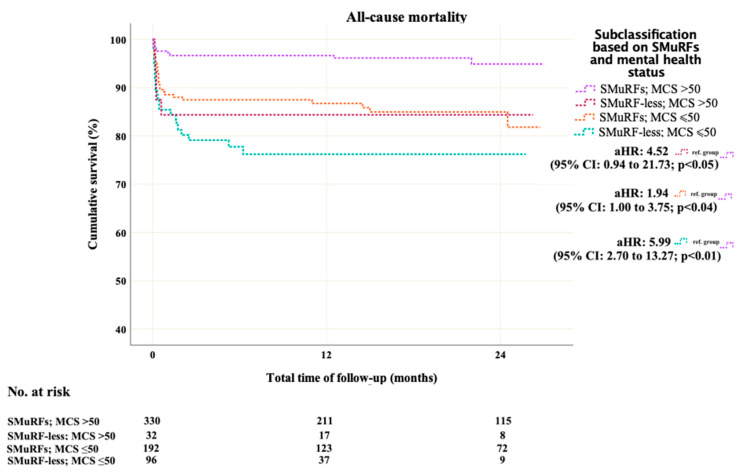
Time-to-event analysis for all-cause mortality according to SMuRF-less and mental status.

A sub-analysis was also performed by dividing patients into four categories according to SMuRF-lessness and mental status. The corresponding time-to-event analysis is depicted in Figure 4. Cox regression analysis is shown in Supplementary Table S1. By setting the patients with SMuRFs and MCS > 50 as a reference group, the aHRs for all-cause death were 5.99 (95% CI: 2.70 to 13.27; *p* < 0.01) for the group of SMuRF-less patients with MCS ≤ 50, 1.94 (95% CI: 1.00 to 3.75; *p* = 0.047) for patients with SMuRFs and MCS ≤ 50, and 4.57 (95% CI: 0.94 to 21.73; *p* = 0.059) for SMuRF-less patients with MCS > 50%.

## 4. Discussion

This post-hoc analysis of the “Beyond-SmuRFs” prospective cohort including patients with AMI showed that poor MHS is independently associated with the occurrence of SMuRF-less AMI, which suggests a potential link between mental distress and the development of AMI in patients without traditional risk factors. This finding aligns with emerging evidence that psychosocial stressors, depression, and anxiety can contribute to cardiovascular events, potentially through mechanisms such as autonomic dysfunction, inflammation, and endothelial dysfunction. Furthermore, impaired mental health is linked to worse outcomes after the index AMI, suggesting that mental status is an independent contributor to worse outcomes, beyond conventional cardiovascular risk factors. It is noteworthy that SMuRF-less patients with AMI suffered higher mortality rates compared to those with SMuRFs. Our study expands the existing literature regarding the correlation between MHS and SMuRF-less AMI.

Several studies and scientific statements have highlighted the association between mental illness and AMI or accelerated atherosclerosis [22,23]. A recent nationwide Korean cohort study demonstrated that young adults with mental disorders had an up to 3-fold elevated risk of AMI or stroke [24]. In general, individuals diagnosed with severe mental illness (i.e., schizophrenia, bipolar disorder, and major depressive disorder) have an elevated risk of CAD development compared with control populations [25]. The mechanistic substrate of the higher risk in these patients is unclear and may be related to an increased co-prevalence of risk factors including metabolic syndrome, smoking and hypertension, with mental distress or disease (presumably because there is a higher tendency towards compulsive unhealthy lifestyle choice), as well as oxidative stress [22]. A recent meta-analysis confirmed that increased markers of oxidative stress can be observed in schizophrenia and non-treated bipolar disorder [26,27]. However, the exact mechanisms remain unexplained. Interdisciplinary mechanistic work combining neuropsychology, cardiovascular stress physiology, and advanced imaging is needed to improve our understanding of these stress pathways, which potentially play a critical role in CAD [28].

**Figure 5:**
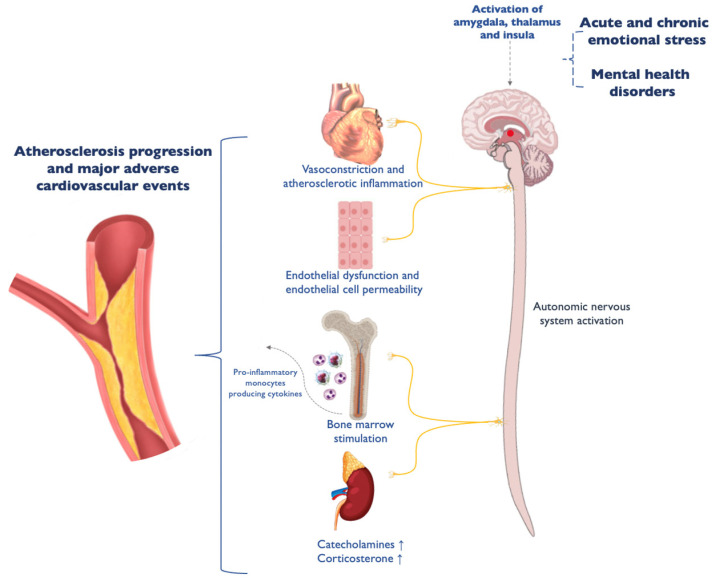
Potential pathophysiological mechanisms of mental-stress-induced myocardial infarction.

Our study expands existing literature regarding the correlation between MHS and AMI, and in particular SMuRF-less AMI, demonstrating such an association among patients not with mental illness or other well defined mental disorders, but merely with a low score in the mental health component of a general QoL questionnaire. A large observational study [29] has demonstrated that psychological distress has a dose-dependent, positive, independent association with the absolute risk of AMI and stroke in both men and women. It is hypothesized that mental distress and CAD could have a shared etiology, including biological, behavioral, psychological, and genetic mechanisms [23]. Recently, a new term has been coined to describe MIs associated with psychological distress—“mental-stress-induced MI” (MSIMI) [11]. This clinical entity seems to be not uncommon among CAD patients [30] and covers a great range of clinical manifestations, including normal atherosclerosis-induced AMI and MI with No Obstructive Coronary Arteries (MINOCA) [11,31]. MSIMI seems to occur at lower oxygen demand than conventional stress ischemia. Other potential underlying mechanisms include endothelial dysfunction, enhanced smooth muscle reactivity, β1-adrenergic receptor genetic susceptibility, microvascular dysfunction, peripheral vasoconstriction, and inflammatory, cortisol, coagulation and hypothalamic pituitary adrenal responses [11,32]. Finally, MSIMI has been also linked with increased activation in the medial and the rostro-medial prefrontal cortex and the inferior frontal cortex, along with other pain processing regions (thalamus, insula, and amygdala) [11,33,34]. Τhese pathogenetic mechanisms are summarized in Figure 5. Furthermore, patients with severe mental illnesses seem also to be at increased risk of missed MI diagnosis since they present more often with silent CAD, they are less commonly treated with proper clinical management, and they face social isolation resulting in the suboptimal management of cardiovascular risk factors [35,36].

Diving further into the pathophysiology, several mechanisms may underlie the increased cardiovascular risk in SMuRF-less AMI patients, extending beyond traditional risk factors. Oxidative stress has been implicated in vascular injury, endothelial dysfunction, and plaque instability in patients with mental disorders. The excessive production of reactive oxygen species leads to reduced nitric oxide availability, impaired vasodilation, and increased thrombogenicity, potentially accelerating atherosclerotic processes even in individuals without classical risk factors [37]. Additionally, endothelial dysfunction, a hallmark of early atherosclerosis, may be present in SMuRF-less AMI patients, driven by impaired vascular repair mechanisms and microvascular dysfunction. This may result in heightened arterial stiffness and reduced coronary reserve, predisposing individuals to acute coronary events despite the absence of conventional risk markers. Moreover, inflammation plays a central role in AMI pathogenesis, with elevated levels of cytokines such as interleukin-6 and tumor necrosis factor-alpha contributing to endothelial activation, monocyte adhesion, and plaque vulnerability. Even in the absence of hypertension or hyperlipidemia, chronic low-grade inflammation—often accompanying psychiatric disorders—can promote coronary events [38]. Autonomic dysfunction is another crucial factor, particularly in individuals with mental health disorders. An imbalance between sympathetic and parasympathetic regulation may contribute to vasospasm, increased myocardial oxygen demand, and a proarrhythmic state, exacerbating cardiovascular risk. Mental distress further amplifies this dysfunction, reinforcing a vicious cycle of stress-related cardiovascular dysregulation [39]. Understanding these alternative pathways not only enhances the clinical recognition of at-risk individuals, but also underscores the importance of integrating non-traditional markers into cardiovascular risk stratification models.

Our study provides evidence of a significant association between poor mental health and all-cause mortality in patients following AMI. These findings suggest that mental factors might play an important role in the prognosis of post-AMI patients, aligning with previous research underscoring the impact of MHS on cardiovascular outcomes, including mortality [8,40]. Several mechanisms may underlie this observed association. Firstly, psychological distress, such as depression and anxiety, has been linked to the dysregulation of the autonomic nervous system, increased inflammation, and endothelial dysfunction [41,42], all of which contribute to the progression of atherosclerosis and adverse cardiovascular events. Secondly, psychological factors may exacerbate the effects of traditional cardiovascular risk factors, such as hypertension, dyslipidemia, and diabetes, further increasing the risk of adverse outcomes [43]

Furthermore, an interesting aspect of our results is that patients with SMuRFs tend to have better outcomes than SMuRF-less patients in the short term. Several studies have reported higher rates of major adverse cardiac events, as well as increased short-term all-cause and cardiovascular mortality in SMuRF-less patients [44,45]. This elevated risk persisted in our prospective trial and other observational studies, even after multivariable adjustments [5,46,47,48,49,50,51,52]. A meta-analysis by Kong et al. highlighted a 60% higher in-hospital mortality rate in SMuRF-less AMI patients compared to those with at least one SMuRF [16]. One possible explanation is the greater prevalence of cardiac arrest and cardiogenic shock at baseline in SMuRF-less cohorts, as seen in our study [47]. Evidence consistently shows they are more likely to experience life-threatening AMI complications, including ventricular arrhythmias [46,47,48]. For example, Kelly et al. found that SMuRF-less patients had over twice the risk of cardiac arrest at presentation, and were more likely to require vasopressors, mechanical support, or intensive care [53]. Additionally, non-traditional factors such as autonomic dysfunction, oxidative stress, environmental influences, and systemic inflammation may contribute to their vulnerable atherosclerotic profile.

In our study, we emphasize the importance of MHS as a key factor in the prognosis of AMI patients, particularly those without traditional cardiovascular risk factors (SMuRF-less). Mental health should be considered in the clinical management of AMI patients, as poor mental health has been shown to independently predict worse outcomes, including all-cause mortality. However, alongside MHS, it is essential to consider established prognostic parameters when assessing the long-term risk for AMI patients. Left ventricular ejection fraction, a well-established predictor of mortality and heart failure, remains crucial for risk stratification [54]. Renal dysfunction, measured by estimated glomerular filtration rate (eGFR), also plays a central role in predicting poor outcomes in AMI survivors [55] Furthermore, in-hospital bleeding has been identified as a significant prognostic element in AMI patients [56]. Factors such as older age, female sex, hypertension, and peripheral artery disease are identified as independent predictors of in-hospital bleeding, while radial access and preserved LVEF provide protective benefits [56,57]. In our study, we show that mental distress, in combination with these traditional prognostic markers, offers a more nuanced understanding of AMI prognosis. As a result, it is crucial to integrate mental health assessments with conventional cardiovascular risk factors to better identify high-risk patients.

These findings indicate the importance of integrating mental health assessment and support into routine clinical care for patients with AMI. Identifying patients with impaired MHS early in the post-AMI period and providing targeted interventions, such as cognitive behavioral therapy, psychoeducation, and pharmacotherapy, may help improve psychological well-being and reduce mortality risk. Future research should focus on elucidating the specific pathways through which MHS influences cardiovascular outcomes and evaluating the effectiveness of interventions targeting mental health in improving prognosis following AMI. However, whether we can really optimize post-MI recovery and reduce the burden of mortality in this high-risk population remains to be proven in clinical trials.

## 5. Limitations

This study should be interpreted in the context of certain recognized limitations.

Despite thorough adjustments for demographic and clinical factors, residual confounding may still be present, particularly regarding the relationship between poor MHS and SMuRF-less AMI. Due to the observational nature of our study, we cannot establish a definitive causal relationship between mental health status and SMuRF-less AMI. It remains uncertain whether poor mental health contributes to the development of AMI or if undiagnosed cardiovascular risk factors were present before the event. Further prospective studies are needed to clarify this temporal association. Moreover, although extensive adjustments for physical condition variables were made, it remains possible that certain physical conditions caused by SMuRFs may have contributed to worse physical health, and were therefore reflected in the mental health assessments. Furthermore, pre-AMI mental status was recorded as a self-reported health metric after AMI, which may introduce reporting bias. Third, the SF-36 score was provided only at initial enrollment; therefore, there were no available data about score changes over time. Furthermore, the threshold of MCS of 50 was established based on previous research [58]; however, no specific threshold analysis or ROC curve analysis has been conducted. Finally, this study population consists of Greek patients exclusively. Future studies should be conducted that include other populations to account for the inherent variability of different patient populations and test the generalizability of our results.

## 6. Conclusions

This analysis of a real-world, prospective cohort including patients with AMI showed that impaired MHS is an independent predictor of the occurrence of SMuRF-less AMI. Moreover, our results reveal a significant association between elevated mortality rates in patients reporting poor mental status prior to the event, as well as in those lacking SMuRFs. These findings contribute to our comprehension of the interplay between the mind and the heart. Simply phrased, these results suggest that mental status (and not only acute “mental stress”) may be an important risk factor for AMI, whose significance is swamped by the more powerful effect of traditional risk factors, and can therefore be more readily recognized in the absence of SMuRFs. However, this study does not address potential underlying mechanisms, which warrant exploration in future studies.

## Figures and Tables

**Table 1:** Baseline characteristics of the study population.

Parameters	MCS > 50 (N = 362)	MCS ≤ 50 (N = 288)	*p*-Value
**Demographics**			
**Male Sex—No (%)**	287 (79.3)	210 (72.9)	0.057
**Age (Years)—Means (±SD)**	62.5 (±12.3)	62.7 (±11.8)	0.517
**BMI (Kg/m^2^)—Means (±SD)**	33.6 (±12.1)	32.8 (±6.4)	0.164
**SMuRF-less patients**	32 (8.8)	96 (33.3)	**<0.001**
**Underlying Diseases—No (%)**			
**Hypertension**	181 (50.0)	148 (51.3)	0.725
**Diabetes Mellitus**	90 (24.9)	70 (24.3)	0.872
**Smoking**	188 (51.9)	137 (47.5)	0.269
**Dyslipidemia**	117 (32.3)	84 (29.1)	0.447
**Vascular Disease**	6 (2)	5 (1.7)	0.938
**CKD Stages 4–5**	8 (2.2)	6 (2)	0.912
**Atrial fibrillation**	10 (3.6)	7 (2.4)	0.792
**Major psychiatric disease**	13 (3.5)	14 (4.8)	0.420
**Acute HF on admission**	7 (1.9)	5 (1.7)	0.853
**AMI subcategory**			
**STEMI**	125 (34.5)	87 (30.2)	0.283
**NSTEMI**	237 (65.5)	201 (69.8)	0.174
**Echocardiographic Findings—Means (±SD)**			
**LVEF (%)**	47.2 (9.6)	46.2 (10.5)	0.096
**GLS (** **-%** **)**	13.0 (4.2)	12.2 (4.5)	0.299
**Laboratory Findings—Means (±SD)**			
**eGFR (mL/min)**	85.6 (26.2)	84.5 (23.1)	0.126
**NT-proBNP**	2922 (530)	3594 (570)	0.089
**High sensitivity Troponin T**	1345 (290)	1236 (214)	0.222

* AMI, acute myocardial infarction; AF, atrial fibrillation; BMI, body mass index; CKD, chronic kidney disease; eGFR, estimated glomerular filtration rate; GLS, global longitudinal index; HF, heart failure; LVEF, left ventricular ejection fraction; MCS, mental component summary; Non-ST-Elevation Myocardial Infarction, NSTEMI; ST-Elevation Myocardial Infarction, STEMI.

## Data Availability

The datasets used and/or analyzed during the current study will be provided by the corresponding author upon reasonable request.

## Supplementary Materials

The following supporting information can be downloaded at: https://www.mdpi.com/article/10.3390/jcm14082645/s1, Table S1: Cox regression analysis for the primary outcome (all-cause mortality).

## Abbreviations

AMI = acute myocardial infarction; SMuRF = standard modifiable risk factors; CAD = coronary artery disease; MCS = mental component summary STEMI = ST-segment elevation myocardial infarction; NSTEMI = Non-ST-segment elevation myocardial infarction.
