# Supplementary material for: Poor Mental Health Status as a Risk Factor and Prognosticator in SMuRF-Less Acute Myocardial Infarction
Source: J Clin Med. 2025 Apr 11;14(8):2645. doi: 10.3390/jcm14082645 (PMC12027916; doi:10.3390/jcm14082645)
Supplement: Supplementary file 1 [file jcm-14-02645-s001.zip › jcm-3526611-supplementary.pdf]

Supplementary Table S1. Cox regression analysis for the primary outcome (all-cause mortality).

	**Adjusted Hazard ratios (95% Cis)**	**P-value**
Age	1.00 (0.98-1.03)	0.556
Gender	1.25 (0.67-2.34)	0.472
BMI	1.00 (0.95-1.04)	0.925
STEMI at presentation	3.13 (1.41-6.96)	0.005
Acute Heart failure at presentation	3.57 (1.04-12.5)	0.044
GFR	0.97 (0.95-0.98)	0.001
Combination of SMuRFs and MCS subcategories		0.001
SMuRF-less; MCS>50 (ref SMuRFs; MCS>50)	4.52 (0.94-21.73)	0.049
SMuRFs; MCS≤50 (ref SMuRFs; MCS>50)	1.94 (1.00-3.75)	0.039
SMuRF-less; MCS≤50 (ref SMuRFs; MCS>50)	5.99 (2.70-13.27)	0.001
